# Soil wettability can be explained by the chemical composition of particle interfaces - An XPS study

**DOI:** 10.1038/srep42877

**Published:** 2017-02-17

**Authors:** Susanne K. Woche, Marc-O. Goebel, Robert Mikutta, Christian Schurig, Matthias Kaestner, Georg Guggenberger, Jörg Bachmann

**Affiliations:** 1 Institute of Soil Science, Leibniz Universität Hannover, Herrenhäuser Str. 2, 30419 Hannover, Germany; 2 Soil Science and Soil Protection, Martin Luther University Halle-Wittenberg, Von-Seckendorff-Platz 3, 06120 Halle (Saale), Germany; 3 Laborgesellschaft für Umweltschutz mbH, Waldheimer Str. 1, 04746 Hartha, Germany; 4 Department for Environmental Biotechnology, Helmholtz Centre for Environmental Research, UFZ, Permoserstr. 15, 04318 Leipzig, Germany

## Abstract

Soil wettability (quantified in terms of contact angle, CA) is crucial for physical, chemical, and biological soil functioning. As the CA is determined by components present within the outmost nanometer of particles, this study applied X-ray photoelectron spectroscopy (XPS) with a maximum analysis depth of 10 nm to test the relationship between CA and surface elemental composition, using soil samples from a chronosequence where CA increased from 0° (0 yrs) to about 98° (120 yrs). Concurrently, as seen by XPS, C and N content increased and the content of O and the mineral-derived cations (Si, Al, K, Na, Ca, Mg, Fe) decreased. The C content was positively correlated with CA and least squares fitting indicated increasing amounts of non-polar C species with soil age. The contents of O and the mineral-derived cations were negatively correlated with CA, suggesting an increasing organic coating of the minerals that progressively masked the underlying mineral phase. The atomic O/C ratio was found to show a close negative relationship with CA, which applied as well to further sample sets of different texture and origin. This suggests the surface O/C ratio to be a general parameter linking surface wettability and surface elemental composition.

One of the main soil processes with implications for physical as well as chemical and biological soil functioning is the interaction between solid and liquid phase. The spatial and temporal pattern of soil solution within the soil profile and thus the extent of transport depend on soil wetting properties, i.e., the behavior of the soil towards a liquid^1^. Wettability usually is quantified by the contact angle (CA) that forms at the three-phase boundary after placing a drop of liquid on a solid surface^2^. A CA of zero describes a wettable (hydrophilic), a CA > 0° and <90° a subcritically water repellent, and a CA ≥ 90° a non-wettable (hydrophobic) surface. Soil water repellency as a common environmental phenomenon^3^ is caused by non-polar organic surfaces of either interstitial particulate organic debris or organic components adsorbed on particle surfaces^4^. Adsorbed inorganic and organic compounds, including biogenic components such as microbial residues, form the biogeochemical interfaces (BGI)^5^,^6^ as the transition zone between mineral surface and pore space with a lateral extension ranging over several magnitudes^7^ including films and layers of varying thickness (nm range) and adsorbed particulate organic matter (OM) (μm range).

Besides liquid transport many other processes contributing to soil functioning such as adsorption and stabilization of OM and pollutants as well as biological interactions rather are controlled by BGI wetting properties than by bulk particle or bulk soil characteristics. The CA is determined by polarity and orientation of the functional groups of the outer 0.5–1 nm of the surface layer, also termed as ‘CA interphase’^8^. In soils non-polar functional groups mostly include C–H bonds (e.g., –CH_3_) while polar functional groups mostly include either an O–H or a C–O bond (e.g., –OH, –COOH), which identifies C, O, and H as the elements mainly involved in particle wettability. Generally, liquid transport will be favored in a matrix with predominantly hydrophilic surfaces and will increasingly be reduced with increasing percentage of hydrophobic surfaces. Heterogeneous distribution of hydrophobic surfaces can result in preferential flow paths on the pedon or macro scale^9^, but these effects are also operative on the particle or micro scale^10^,^11^. Under saturated conditions, deposition of negatively charged model colloids was found to be enhanced on hydrophobized quartz surfaces as compared to their hydrophilic counterparts^12^, emphasizing the role of soil wetting properties for colloidal retention and groundwater quality. Reduced wettability is also considered to favor OM stabilization^13^ and aggregate stability^14^. A negative impact on crop yield seems indicated for even low degrees of water repellency^15^ and the occurrence of soil water repellency is suggested to be fostered by global warming and more frequent droughts^16^.

BGI contribute only minor to total soil mass and volume and their specific characteristics will not be revealed by bulk soil analysis^7^. Given that wettability is determined by the components of the CA interphase (i.e., the outmost BGI layer), it should be strongly related to the CA interphase’s elemental composition. However, common surface analytical techniques such as energy dispersive spectroscopy (EDS) and reflectance infrared spectroscopy (diffuse reflectance infrared Fourier transform spectroscopy, DRIFT; attenuated total reflection Fourier transform infrared spectroscopy, ATR-FTIR) probe a surface layer in the μm range, which limits the detection of components specific for the outmost surface layer. A promising tool to confine the analysis depth to the upper nanometers is X-ray photoelectron spectroscopy (XPS). Here, the surface is irradiated by X-rays, resulting in the emission of photoelectrons (PE). Evaluation of the element-specific binding energy (BE) of the PE allows an element analysis including all elements with an atomic number of Z > 2. Due to the mean free path (*λ*) of PE in solid matter (0.5–3 nm in the energy range studied), the collected PE originate from a 2–10 nm thin surface layer, i.e., the XPS analysis depth is similar to CA analysis depth, which might allow to link wetting properties and surface elemental composition with higher accuracy than achievable with EDS and reflectance infrared techniques. Previous studies showed that XPS-based element information could explain changes in wetting properties during aging of wood^17^,^18^ and after plasma-treatment of polymer surfaces^19^, or the CA hysteresis of a crosslinked polymer surface^20^.

In natural systems, BGI are formed during soil development where mineral weathering as well as increasing participation of biological processes turn rock into soil^21^. Early to intermediate stages of soil chronosequences^22^ offer a unique approach to study initiation and development of BGI. Here, mineralogical composition and texture undergo only minor changes while along with the input of plant and microbial residues and development of soil OM physical and chemical soil particle properties are extensively modified. Schurig *et al*. for example showed by scanning electron microscope analysis an increasing coverage of mineral particle surfaces by microbial cell envelope fragments from initial stages of soil formation to a soil age of 120 yrs in soils from the Damma glacier chronosequence (Switzerland)^23^. At the same time soil wetting properties distinctly changed as CA increased from 0° (0 yrs) to around 98° (120 yrs). The close correlation between the extent of coverage by bacterial cell envelope fragments (containing aminosugars, proteins, and lipids) and CA^23^ along with the findings of Achtenhagen *et al*. that water- and salt-stressed bacteria on mineral surfaces exhibit increased CA^24^ suggested an impact of bacterial necromass on particle wetting properties.

The distinct relationship between wetting properties and BGI development at the Damma glacier soil chronosequence calls at a more detailed analysis of particle surface elemental composition particularly with respect to the CA interphase. The main objective of this study, therefore, was to utilize XPS to determine the surface elemental composition of the soil particles as function of soil age and to relate the surface chemical information to CA. We hypothesize that XPS with a maximum analysis depth close to CA analysis depth is capable of directly linking modifications in surface elemental composition and changes in wetting properties. Using the obtained data as a base, we will test if addition of further data from various materials permits to derive a generally valid chemical proxy that allows prediction of soil wetting properties independent of kind and origin of the material regarded.

## Results and Discussion

### Surface Elemental Composition

Apart from C and N, the elemental composition of the particle surface layer reflected the granitic composition with quartz, feldspars, and micas as main components^25^. Beside O as the most abundant element, Na, Fe, N, K, Ca, C, Si, Mg, and Al could be identified for all soil ages (Table 1 and Supplementary Fig. S1). As function of soil age, i.e., with progressing soil and BGI development, surface elemental composition showed characteristic changes: Over the whole period of time (0–120 yrs) the content of O and the mineral-derived cations generally decreased and the content of both C and N distinctly increased. While C content increased continuously from 0 to 110 yrs by an overall factor of about 2.5, N content reached a maximum (about fourfold) after 80 yrs and then slightly decreased at 120 yrs (Table 1 and Supplementary Fig. S2). With increasing soil age additionally traces of P could be identified.

### Relationship between Surface Elemental Composition and Wetting Properties

**Figure 1:**
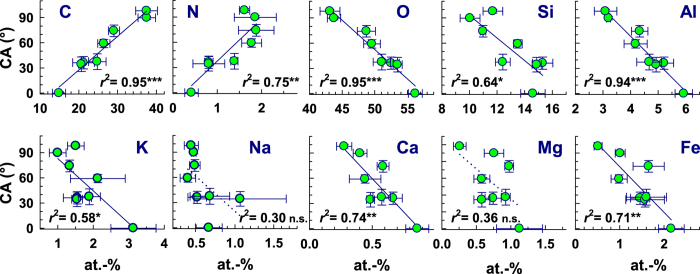
Surface wetting properties in terms of contact angle (CA) as function of XPS-detected element content. The lines represent linear regression fits. Significance levels: *P < 0.05; **P < 0.01; ***P < 0.001. The dotted lines added to the plots with no significant correlation (Na, Mg) indicate the general trend.

The distinct change of soil wetting properties with soil age from wettable (0 yrs) to subcritically water repellent (7, 15, 65, 70, 80, 110 yrs) and finally hydrophobic (120 yrs; Table 1) had been found to be closely positively related to bulk C content (*r*^2^ = 0.87)^23^. As expected, the correlation was even closer with surface C content (*r*^2^ = 0.95, *P* < 0.001; Fig. 1). The decrease in O almost mirrored the increase in C and accordingly CA was negatively correlated with O (*r*^2^ = 0.95, *P* < 0.001; Fig. 1). Further, a negative correlation was found between CA and Al (*r*^2^ = 0.94, *P* < 0.001) and, to a lesser extent, Ca (*r*^2^ = 0.74, *P* < 0.01), Fe (*r*^2^ = 0.71, *P* < 0.01), Si (*r*^2^ = 0.64, *P* < 0.05), and K (*r*^2^ = 0.58, *P* < 0.05). The relations between CA and Mg (*r*^2^ = 0.36, *P* = 0.118) and Na (*r*^2^ = 0.30, *P* = 0.158) were weakest and not significant (Fig. 1). The generally weaker relationship exhibited by the charge-compensating and octahedral cations (Na, K, Ca, Fe, Mg) with either soil age and CA may have been caused by a non-stoichiometric distribution of these elements at the mineral surface due to weathering^26^. In contrast, the noticeable close relationship between CA and Al may have been caused by the high affinity of OM for Al-bearing phases such as alumosilicates and Al (hydr)oxides^27^ that resulted in preferred attachment to these sites. Despite the maximum N content between 80 and 110 yrs still a significant positive relationship with CA was observed (*r*^2^ = 0.75, *P* < 0.01) (Fig. 1).

The distinct increase in C and N content with soil age largely corresponded to the results from bulk analysis^23^. For the youngest soil (0 yrs), bulk analysis revealed only small amounts of C and N (<0.05 wt.-%)^23^. XPS analysis confirmed that for N but indicated C as the most abundant element besides O (about 15 at.-%; Table 1). This may be due to C components released during deglaciation and attached to mineral surfaces^28^ and microbial cell envelope fragments found on quartz surfaces already for the youngest soil^23^. Additionally, in general, a certain contribution of adventitious C, i.e., C compounds adsorbed to the surface during contact with ambient air cannot be excluded^29^. As all C components present contribute to particle wetting properties, no attempt was made to specify the proportion of adventitious carbon.

The decrease in surface cation content with soil age seemed contrary to bulk soil element contents determined by Bernasconi *et al*.^25^ for the same study sites and a similar soil depth (5–10 cm, Supplementary Table S1). Here no distinct pattern of cation content as function of soil age was observed. At bulk soil basis, the chemical index of alteration [CIA = Al/∑ (Na, K, Ca, Mg, Al)]^25^ was comparable to that of the fresh rock over the whole chronosequence (0.55–0.65), suggesting a minor degree of chemical weathering^25^. The XPS-based CIA was similar (about 0.55) and due to the general decreasing cation content with increasing soil age also showed no defined shift with soil age (Table 1). The decreasing cation content therefore indicates an increasing surface coating by organic compounds during BGI formation that reduced the amount of underlying mineral phase within XPS analysis depth with increasing soil age. A surface coverage by organic material was already assumed by Flogeac *et al*. to explain the only low surface Fe content of ferric iron oxides^27^. The strong negative correlation between C and especially Al (*r*^2^ = 0.97, *P* < 0.001) and to a lesser extent Si (*r*^2^ = 0.73, *P* < 0.01), Ca (*r*^2^ = 0.71, *P* < 0.01), Fe (*r*^2^ = 0.70, *P* < 0.01), and K (*r*^2^ = 0.51, *P* < 0.05; Supplementary Fig. S3) may support this assumption and would explain the negative correlation between the mineral-derived cations and CA. Likewise, these findings may indicate a coating consisting predominantly of C and N compounds only little associated with metals. The BE of N around 400 eV for all soil ages also points to organic compounds as N in mineral form has higher BE of 401–407 eV^30^. According to Pantano & Wittberg^31^ the layer thickness *t* (nm) of a coating can be determined from the PE mean free path *λ* (nm) of an element *X* occurring only in the underlying material but being still detected in the spectra of the coated surface and the content of *X* determined for the non-coated and the coated surface. The content of *X* of the non-coated surface is derived from regression analysis using a PE with similar *λ* of an element *Y* occurring only in the coating (for details see Supplementary). This approach ideally assumes a homogeneous, continuous coating. Calculated values for non-ideal surfaces like soil particles thus will represent an average layer thickness that includes thin films as well as attached particulate OM^30^. We calculated mean layer thicknesses *t (n* = 6) from Si (*X*_1_; Si 2p; *λ* = 3.2 nm; *t*_Si_) and Al (*X*_2_; Al 2p; *λ* = 3.3 nm; *t*_Al_) as the two main components of the silicate-dominated soil matrix and C (*Y*; C 1 s; *λ* = 3.0 nm) as the main coating component. A close positive correlation of *t* was found with soil age (*r*^2^ = 0.95, *P* < 0.001, Supplementary Fig. S4) while over the whole period of time (0–120 yrs), in agreement with the detection of the mineral-derived cations in all spectra, a layer thickness well below 10 nm was indicated with an overall increase in *t* by a factor of about 3 (Table 1). In agreement with the detected C content in the youngest soil (0 yrs) the presence of a very thin organic layer was indicated, as regression analysis indicated higher Si and Al contents than actually measured (see Supplementary Fig. S3). A close and significant positive correlation of *t* was observed with CA (*r*^2^ = 0.87, *P* < 0.001; Supplementary Fig. S5). The very close and significant positive relationship of *t* with C (*r*^2^ = 0.96, *P* < 0.001) and the still significant positive relationship of *t* with N (*r*^2^ = 0.74, *P* < 0.01; Supplementary Fig. S6) also indicates OM as the main coating component.

**Figure 2:**
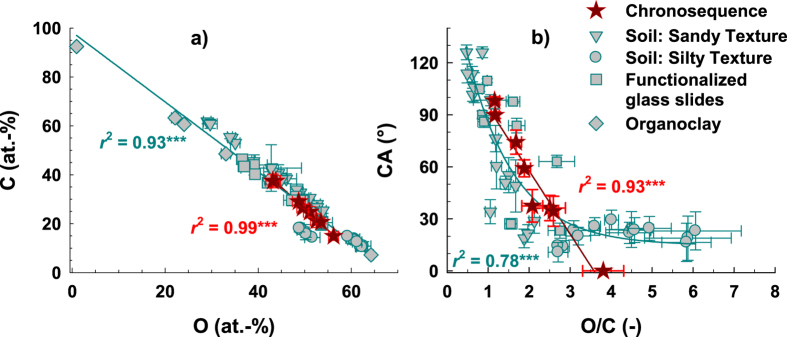
Relationship between surface O and C content (a, *n* = 55) and contact angle (CA) as function of the surface atomic O/C ratio (b, *n* = 48) for the chronosequence samples (red stars) and further sample sets differentiated by texture and origin (grey symbols). The organoclay, i.e., the suite of bentonite samples treated with different amounts of hexadecylpyridiniumchloride^37^, only is shown in the left graph. The lines represent linear and exponential regression fits. The coefficients of dtermination and significance levels in red refer to the chronosequence samples and the coefficients of determination and significance levels in cyan refer to all sample sets including the chronosequence samples. Significance level: ***P < 0.001.

Oxygen was closely negatively correlated with CA (Fig. 1) and, hence, as well with C (*r*^2^ = 0.99, *P* < 0.001; Fig. 2a). Accordingly, the atomic O/C ratio showed a close negative relationship with CA (*r*^2^ = 0.95, *P* < 0.001; Fig. 2b and Table 1). A correlation between surface O/C ratio and wetting properties already was observed for other materials, e.g., wood^17^,^18^ and differently treated polymer surfaces^19^,^32^,^33^. For soil samples exposed to elevated temperatures (40–105 °C) Diehl *et al*. observed along with increasing CA a decrease in surface O/C ratio^34^ and for a soil transect testing the influence of stemflow infiltration on CA, lower O/C ratios were associated with higher CA^35^. However, while in Diehl *et al*. all CA were >90°^34^ and in Krueger *et al*. all CA were <90°^35^, the present study demonstrates the close correlation between CA and surface O/C ratio for the whole CA range from 0° to >90°, suggesting a general relationship. The combination of our chronosequence data, the data of Diehl *et al*.^34^, Krueger *et al*.^35^, and Pronk *et al*.^36^ and data of further soil sample sets and functionalized glass surfaces (unpublished results) could be well described by an exponential function, indicating indeed a close and general relationship between CA and surface atomic O/C ratio (*r*^2^ = 0.78, *P* < 0.001, *n* = 48; Fig. 2b). An O/C ratio of about unity divides hydrophobic (CA ≥ 90°) from subcritically water repellent and hydrophilic surfaces (CA < 90°). An O/C ratio >3 roughly includes material with only small initial CA < 30° and hydrophilic material. Some influence of texture is indicated as samples with sandy texture and CA from 0° to >90° cover a wide range of O/C ratios from about 4 to <1, while silty material generally is characterized by small CA and high O/C ratios of >3. So, at least for material consisting of inorganic solid matter coated by organic components (i.e., mineral surfaces with adsorbed OM or functionalized glass surfaces) these findings suggest the surface atomic O/C ratio to be a general parameter to relate wetting properties and surface elemental composition. To the best of our knowledge, no study so far could demonstrate this relationship for CA in the range from 0° to around 120° independent of surface structure as the samples tested included smooth as well as rough surfaces. This at the same time demonstrates that surface roughness seems not to challenge quantification, at least with respect to the main compounds (for a brief discussion on surface roughness see Supplementary).

The assumption of generality is further supported by the close correlation between C and O that was not restricted to the chronosequence samples but was found for all samples tested, including an organoclay with different organic cation loadings^37^ (*r*^2^ = 0.93, *P* < 0.001, *n* = 55; Fig. 2a). However, it remains to be tested if this relationship and especially the validity of the surface O/C ratio as a proxy for wetting properties also holds true for pure homogeneous materials that do not exhibit a surface coating like, e.g., biopolymers or microorganisms.

**Figure 3:**
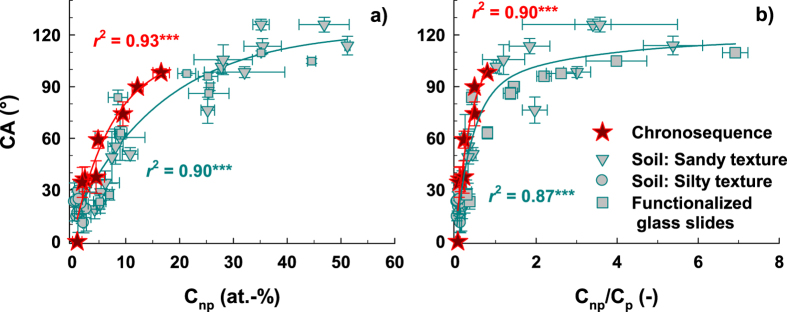
Contact angle (CA) as function of the amount of non-polar C species (C_np_) (a; *n* = 48) and as function of the ratio between non-polar and polar C species (C_np_/C_p_) (b; *n* = 48) for the chronosequence samples (red stars) and further sample sets differentiated by texture and origin (grey symbols). The lines represent exponential regression fits. The coefficients of determination and significance levels in red refer to the chronosequence samples and the coefficients of determination and significance levels in cyan refer to all sample sets including the chronosequence samples. Significance level: ***P < 0.001.

In contrast to the mineral-derived cations, O is a constituent of both soil minerals and OM. Following the approach of Brodowski *et al*.^38^, O bound to C (O_C_) can be differentiated from O bound to mineral-derived cations (O_cation_) according to


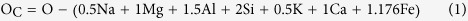


(element contents in at.-%). Application of the algorithm indicated for the chronosequence samples a roughly constant O_C_ content over the whole period of time (120 yrs, Table 1) and accordingly O_C_ only showed a very weak and not significant positive correlation with C (*r*^2^ = 0.39, *P* = 0.100) and with CA (*r*^2^ = 0.33, *P* = 0.135). In contrast, O_cation_ was negatively correlated with C (*r*^2^ = 0.95, *P* < 0.001) as well as with soil age (*r*^2^ = 0.95, *P* < 0.001) and CA (*r*^2^ = 0.88, *P* < 0.001; Supplementary Figs. 7, 8, 9). In line with the observed wetting properties (i.e., increasing CA and increasing CA stability, Table 1) and the nearly constant O_C_ content, this may indicate less polar and more non-polar C species within the newly acquired OM on mineral surfaces with increasing soil age. Applying a fitting scheme for the C 1 s peak with two sub-peaks representing polar (C_p_) and non-polar (C_np_) C species^35^,^36^ indeed indicated distinct changes in OM composition with soil age (Table 1). While for the youngest soil (0 yrs) >90% of total C was polar (C_np_/C_p_ = 0.1; Table 1), for the oldest soil (120 yrs) the amount of C_np_ was >40% of total C (C_np_/C_p_ = 0.8; Table 1). With increasing soil age the C_p_ content increased from 0 to 7 yrs and then up to 120 yrs showed some fluctuations. In contrast, the C_np_ content increased constantly from 0 to 120 yrs (Table 1 and Supplementary Fig. S10), matching the increase in phospholipid fatty acid and total lipid fatty acid amounts^23^. Consequently, in line with Krueger *et al*.^35^, CA and C_p_ showed a very weak and not significant positive correlation (*r*^2^ = 0.36, *P* = 0.119). This may be explained by the fact that the presence of only polar C species should always result in a wettable (hydrophilic) surface^36^. Reduced wettability then should be a function of the amount of non-polar C species as was already assumed by Krueger *et al*.^35^. Indeed, correlation between CA and C_np_ was very close and significant (*r*^2^ = 0.91, *P* < 0.001; Fig. 3a). Application of the fitting scheme to the sample sets displayed in Fig. 2 revealed a very close positive correlation between CA and C_np_ independent of texture and kind of surface (*r*^2^ = 0.90, *P* < 0.001, *n* = 48; Fig. 3a). Several DRIFT studies tried to explain the observed wetting properties by the A/B ratio (i.e., the ratio of the absorption intensity of the non-polar (A) and polar (B) functional groups)^39^,^40^,^41^. No general relationship, however, could be derived, probably due to the considerably larger depth probed by DRIFT spectroscopy. In contrast, the XPS-derived C_np_/C_p_ ratio showed a close and significant positive relationship with CA for the chronosequence samples (*r*^2^ = 0.90, *P* < 0.001) as well as for all sample sets tested (*r*^2^ = 0.86, *P* < 0.001, *n* = 48; Fig. 3b). Roughly, a C_p_/C_np_ ratio of about unity divides between CA > 90° and CA < 90°, suggesting that at least half of total C species present has to be non-polar to create a hydrophobic surface.

The present study benefitted from the unique suite of soil samples virtually differing only in soil age and particle surface elemental composition that allowed observing and quantifying BGI formation. XPS with an analysis depth close to CA analysis depth, in fact, proved to be a suitable tool to link surface elemental composition and CA as was hypothesized. Especially in cases with extremely thin coatings (i.e., distinctly <10 nm; Table 1) the contribution of bulk signals must be minimal to enable detection of even subtle changes within the outmost BGI layer. Earlier XPS studies on soil material mainly tested the surface enrichment or depletion of elements^30^,^42^,^43^,^44^,^45^ or characterized the surface elemental composition^27^,^42^. Our study further linked C (and N) enrichment and resulting depletion of mineral-derived cations within XPS analysis depth to the wetting properties as an important soil physical property that is relevant not only on the pedon but as well on the particle scale with distinct implications for physical (e.g., liquid transport), chemical (e.g., sorption processes), and biological (e.g., microbial respiration) soil functioning. Finally, our study signifies the relationship between CA and surface O/C ratio to be valid for a broad range of surface types ranging from ideal smooth glass surfaces to irregularly shaped rough soil particles of various sizes.

## Experimental

### Material

Parent material of the soil chronosequence is granite overprinted by Alpine low-grade metamorphism with quartz, feldspar, and mica as main components^25^. Soil texture is silty sand, the soil type according to U.S. Soil Taxonomy^46^ a Typic Cryothent^25^. The Damma glacier forefield was sampled in direct proximity to the BigLink project sites (BigLink project, http://www.cces.ethz.ch/projects/clench/BigLink25) below the root layer from a depth of 5–20 cm in order to avoid rhizosphere effects on microbial abundances^23^. The distance to the Damma glacier ranged from 5 to 710 m corresponding to soil ages from 0 to 120 yrs (Table 1). All analyses were made on material <2 mm. Details on sampling procedure and determination of bulk C and N content are reported in Schurig *et al*.^23^. For CA and XPS measurements the same sample aliquots were used. Prior to CA determination the samples were dried over oversaturated CaCl_2_ solution (32% *rH*) until constant weight and subsequently stored in standard laboratory polyethylene containers at room temperature (20 °C). The soil age designations used in this study were the same as in Schurig *et al*.^23^.

### Contact Angle Determination

The wetting properties in terms of CA were determined with the sessile drop method (SDM) using a CCD-equipped CA microscope (OCA 15, DataPhysics, Filderstadt, Germany)^12^. Evaluated was the initial CA directly after placing the drop and ending of mechanical disturbances (denoted as CA^23^) as the mean of six individual drops (12 CA readings). As an estimate of CA stability, CA was determined additionally after 5 seconds (denoted as CA_5s_^47^).

### X-Ray Photoelectron Spectroscopy (XPS)

For XPS measurements the samples were fixed on a sample bar using carbon conductive tape (Agar Scientific Elektron Technology UK Ltd., Stansted, UK). The area of each sample was approximately 0.5 cm^2^. XPS spectra were recorded with an Axis Ultra DLD device (Kratos Analytical, Manchester, UK) using monochromated Al*K*_*α*_ radiation (1486.6 eV; emission current: 20 mA, HV: 6 kV). Survey spectra (binding energy, BE, range: 1200–0 eV) were recorded with a pass energy of 160 eV, a dwell time of 500 ms, and three sweeps per measurement cycle. The resolution was 1 eV and the take-off angle 0°. During measurement the UHV was 4 × 10^–7^ Pa. Three spectra were recorded per sample at three different spots with each measured area in the slot modus comprising 300 × 700 μm. To compensate for electrostatic charging, the surface was flooded with low energy electrons. However, complete compensation was not possible and the BE of all spectra had to be corrected. Assuming that Si only occurred in a tetrahedral Si–O bond within rock-derived silicates that did not undergo any changes as function of soil age we decided to correct for the Si 2p BE of quartz (103 eV^48^). The spectra were quantified using the software Vision 2 (Kratos Analytical, Manchester, UK) and the surface elemental composition evaluated in terms of atomic-% (at.-%) using the relative sensitivity factors implemented in the software. The average coefficient of variation for analyzed element contents based on three replicate measurements was 14%.

Usually the sum C 1 s peak is deconvoluted for C speciation into different bonding forms (e.g., C–H, C–C, C–N, C–O^44^). The present work used a more general approach to characterize the C components present by defining two sub-peaks representative of polar C species (C_p_, BE 285 ± 0.2 eV, e.g., C–O, C = O) and non-polar C species (C_np_, BE 284 ± 0.2 eV, e.g., C–H, C = H, C–N)^35^,^36^. This strategy allows comparison of different samples independent of the specific kind of polar and non-polar C species. The fitting scheme assumed a Gauss distribution for peak shape. The difference in BE of C_p_ and C_np_ was fixed to 1 eV. To account for several C species contributing to the defined peaks, an asymmetric peak shape was allowed. Prior to least squares fitting, the background (as given by Vision 2) was subtracted. For peak fitting the software eXPFit (Version 1.5 for Excel 2007, rogermnix@yahoo.com) was used.

We are aware that sensu stricto XPS analysis (like CA analysis) requires smooth homogeneous surfaces as surface roughness can affect the intensity of the XPS signal^49^,^50^. As an estimate for the influence of surface roughness the full width at half maximum (FWHM) of the Si 2p PE was used. The values of smooth and rough surfaces were similar (around 3 eV, see Supplementary) thus indicating surface roughness not to bias quantification. As well, XPS successfully has been applied to soil particles before to quantify surface elemental composition^27^,^30^,^34^,^35^,^36^,^37^,^42^,^43^,^44^,^45^. In case of the chronosequence, the textural and mineralogical similarity of the soil material studied justifies comparison of the samples and to focus on the effect of soil age only.

### Statistics

The relationship between two parameters was evaluated by linear and exponential regression analysis and calculation of Pearson’s correlation coefficient, *r*, using Sigma Plot 11.0 (Systat Software Inc., San Jose, USA). Statistical significance (*P*-value) was tested by one-way univariate analysis of variance (ANOVA). The *P*-level to reject was 0.05.

## Additional Information

**How to cite this article:** Woche, S. K. *et al*. Soil wettability can be explained by the chemical composition of particle interfaces - An XPS study. *Sci. Rep.* **7**, 42877; doi: 10.1038/srep42877 (2017).

**Publisher's note:** Springer Nature remains neutral with regard to jurisdictional claims in published maps and institutional affiliations.

## Supplementary Material

Supplementary Information

## Figures and Tables

**Table 1:** Surface elemental composition and results of least squares fitting of the C 1 s peak.

	Distance to glacier (m)															
	5		55		110		255		375		515		620		710	
	Soil age (yrs)															
	0		7		15		65		70		80		110		120	
(at.-%)																
Na	**0.7**	*0.2*	**0.5**	*0.1*	**1.1**	*0.6*	**0.7**	*0.3*	**0.4**	*0.1*	**0.5**	*0.1*	**0.5**	*0.0*	**0.4**	*0.1*
Fe	**2.2**	*0.3*	**1.5**	*0.2*	**1.6**	*0.4*	**1.6**	*0.5*	**1.0**	*0.2*	**1.7**	*0.4*	**1.0**	*0.1*	**0.5**	*0.1*
O	**56.2**	*1.1*	**52.5**	*1.2*	**53.5**	*0.7*	**51.1**	*1.3*	**49.6**	*1.3*	**48.7**	*1.4*	**43.8**	*1.0*	**43.1**	*1.5*
N	**0.4**	*0.2*	**0.8**	*0.1*	**0.8**	*0.4*	**1.4**	*0.1*	**1.8**	*0.2*	**1.9**	*0.4*	**1.9**	*0.5*	**1.6**	*0.1*
K	**3.1**	*0.6*	**1.6**	*0.4*	**1.5**	*0.2*	**1.9**	*0.3*	**2.1**	*0.8*	**1.3**	*0.1*	**1.0**	*0.2*	**1.5**	*0.2*
Ca	**0.9**	*0.1*	**0.7**	*0.1*	**0.5**	*0.0*	**0.6**	*0.1*	**0.4**	*0.1*	**0.6**	*0.0*	**0.4**	*0.1*	**0.3**	*0.1*
C	**14.9**	*1.7*	**21.2**	*2.8*	**20.7**	*0.7*	**24.8**	*2.2*	**26.4**	*1.6*	**29.1**	*1.4*	**37.5**	*2.2*	**37.5**	*2.8*
Si	**14.6**	*0.9*	**15.3**	*0.7*	**14.9**	*0.3*	**12.4**	*0.5*	**13.5**	*0.3*	**11.0**	*0.2*	**10.0**	*0.7*	**11.7**	*0.7*
Mg	**1.1**	*0.3*	**0.7**	*0.1*	**0.6**	*0.1*	**0.9**	*0.1*	**0.6**	*0.2*	**1.0**	*0.1*	**0.8**	*0.1*	**0.3**	*0.1*
Al	**5.9**	*0.3*	**5.2**	*0.3*	**4.9**	*0.0*	**4.7**	*0.5*	**4.2**	*0.4*	**4.3**	*0.3*	**3.2**	*0.1*	**3.1**	*0.4*
O_C_	**11.7**	*0.4*	**9.9**	*1.2*	**12.1**	*0.7*	**14.5**	*0.1*	**12.8**	*2.0*	**15.8**	*1.0*	**15.8**	*1.0*	**13.0**	*1.5*
O_cation_	**44.5**	*1.5*	**42.6**	*2.2*	**41.4**	*0.4*	**36.5**	*1.4*	**36.7**	*1.5*	**32.9**	*0.4*	**28.0**	*1.6*	**30.2**	*2.2*
(−)																
C/N	**38.1**	*11.2*	**26.3**	*3.6*	**29.4**	*13.4*	**17.8**	*0.8*	**15.0**	*1.7*	**15.9**	*3.0*	**21.2**	*6.1*	**23.3**	*2.9*
CIA	**0.5**	*0.0*	**0.6**	*0.0*	**0.6**	*0.0*	**0.5**	*0.0*	**0.5**	*0.0*	**0.6**	*0.0*	**0.6**	*0.0*	**0.6**	*0.0*
O/C	**3.8**	*0.5*	**2.5**	*0.4*	**2.6**	*0.1*	**2.1**	*0.3*	**1.9**	*0.2*	**1.7**	*0.1*	**1.2**	*0.1*	**1.2**	*0.1*
(nm)																
*t*	**0.54**	*0.12*	**0.61**	*0.25*	**0.71**	*0.24*	**1.00**	*0.27*	**1.01**	*0.37*	**1.22**	*0.26*	**1.65**	*0.43*	**1.50**	*0.44*
	(at.-%)																
C_p_	**13.9**	*0.9*	**18.8**	*2.9*	**18.7**	*0.7*	**20.3**	*0.7*	**21.6**	*1.0*	**19.6**	*0.9*	**25.3**	*1.5*	**20.9**	*1.9*	
C_np_	**1.0**	*0.9*	**2.4**	*0.1*	**1.9**	*0.0*	**4.5**	*1.6*	**4.9**	*0.6*	**9.5**	*1.2*	**12.2**	*1.0*	**16.6**	*1.5*	
(−)																	
C_np_/C_p_	**0.1**	*0.1*	**0.1**	*0.0*	**0.1**	*0.0*	**0.2**	*0.1*	**0.2**	*0.0*	**0.5**	*0.1*	**0.5**	*0.0*	**0.8**	*0.1*	
(°)																	
CA	**0.0**	*0.0*	**36.3**	*7.3*	**34.4**	*8.7*	**37.5**	*9.4*	**59.2**	*4.9*	**74.2**	*6.9*	**89.8**	*1.9*	**98.1**	*2.3*	
CA_5s_	**0.0**	*0.0*	**13.7**	*12.5*	**7.2**	*11.2*	**17.3**	*11.1*	**33.8**	*9.0*	**45.0**	*10.3*	**74.8**	*6.4*	**85.0**	*2.7*	

O_C_ is the share of O bound to C^38^, O_cation_ is the share of O bound to mineral-derived cations, CIA is the chemical index of alteration^25^, *t* is the coating thickness (determined after Pantano & Wittberg^31^), C_p_ is the share of polar C species, C_np_ is the share of non-polar C species^35^,^36^, CA is the initial contact angle and CA_5s_ is the CA 5 seconds after placement of the water drop. CA_5s_ serves as an estimate about CA stability^47^. The numbers in italics give the standard deviation (*n* = 3, for *t: n* = 6).
