# Supplementary material for: Soil wettability can be explained by the chemical composition of particle interfaces - An XPS study
Source: Sci Rep. 2017 Feb 17;7:42877. doi: 10.1038/srep42877 (PMC5314406; doi:10.1038/srep42877)
Supplement: Supplementary Information [file srep42877-s1.pdf]

<u>**Supplementary Information**</u>

Soil wettability can be explained by the chemical composition of particle interfaces - An XPS study

Susanne K. Woche[*,1], Marc-O. Goebel[1], Robert Mikutta[2], Christian Schurig[3], Matthias Kaestner[4], Georg Guggenberger[1], and Jörg Bachmann[1]

[1]Institute of Soil Science, Leibniz Universität Hannover, Herrenhäuser Str. 2, 30419 Hannover, Germany

[2]Soil Science and Soil Protection, Martin Luther University Halle-Wittenberg, Von-Seckendorff-Platz 3, 06120 Halle (Saale), Germany

[3]Laborgesellschaft für Umweltschutz mbH, Waldheimer Str. 1, 04746 Hartha, Germany

[4]Department for Environmental Biotechnology, Helmholtz Centre for Environmental Research, UFZ, Permoserstr. 15, 04318 Leipzig, Germany

Corresponding author

*e-mail: woche@ifbk.uni-hannover.de, Phone: ++49-511-762 5698

XPS survey spectrum

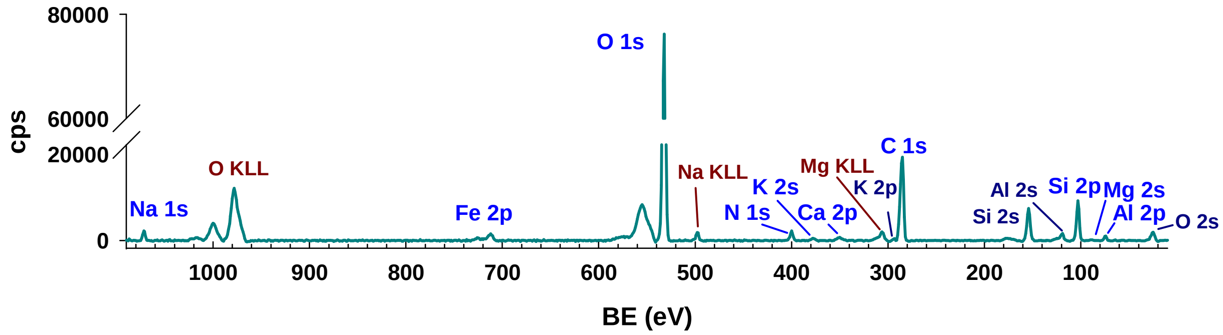

Figure S1. XPS survey spectrum with all elements and photoelectrons (PE) detected marked. Additionally, traces of P could be identified with increasing soil age (P 2s around 190 eV and P 2p around 130 eV). The background (as given by Vision 2, Kratos Analytical, Manchester, UK) has been subtracted. The spectrum was corrected to the Si 2p binding energy of quartz (BE; 103 eV[1]). The PE marked in light blue were used for quantification. Additionally the Auger electrons are indicated (dark red).

Surface elemental composition as function of soil age

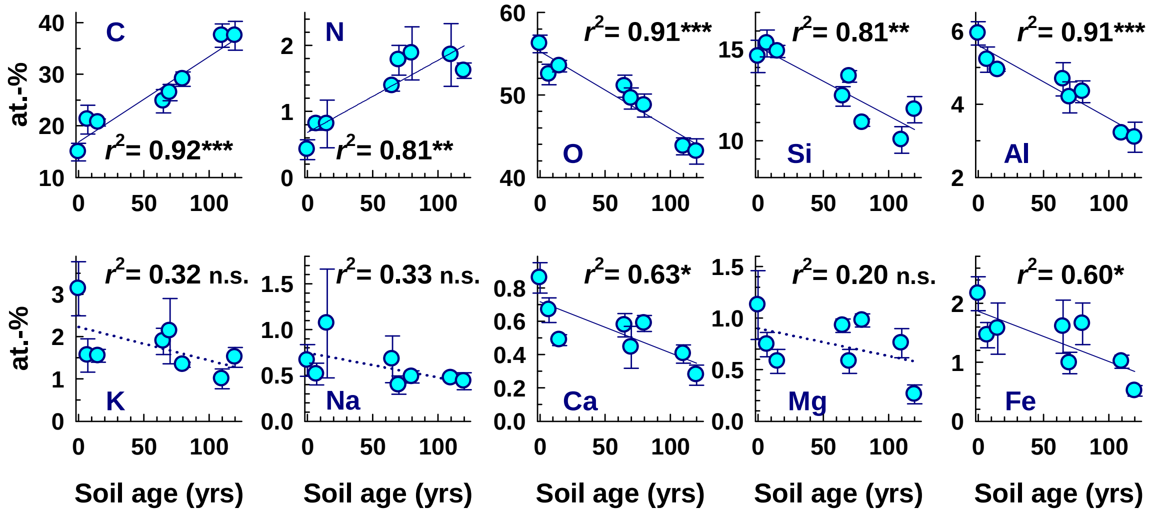

Figure S2. Surface element contents as function of soil age. The lines represent linear regression fits. Significance levels: * $P < 0.05$; ** $P < 0.01$; *** $P < 0.001$. The dotted line added to the plots with no significant correlation (K, Na, Mg) indicates the general trend.

Relationship between C and mineral-derived cations

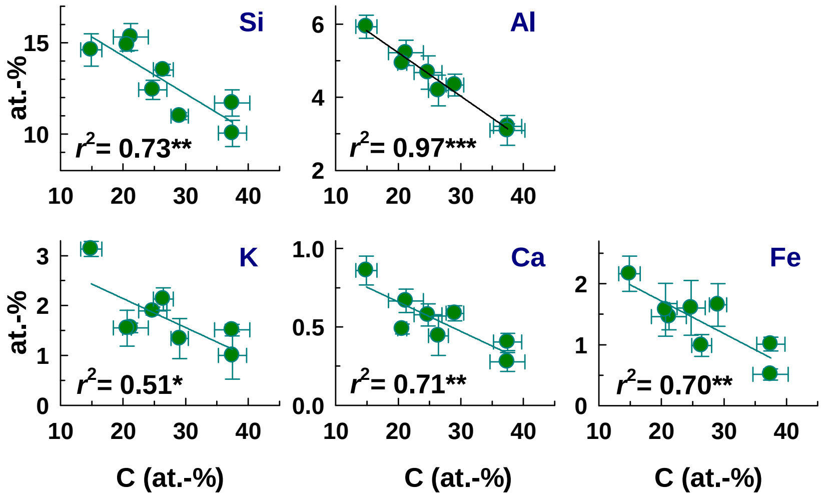

Figure S3. Relationship between the contents of mineral-derived cations and C content. The lines represent linear regression fits. Significance levels: * P < 0.05; ** P < 0.01; *** P < 0.001. The correlation between C content and Mg and Na content was not significant (Mg: r^2 = 0.30, P = 0.161; Na: r^2 = 0.30, P = 0.164).

Bulk element content as function of soil age

As an estimate for the bulk element contents as function of soil age bulk analysis data from the study of Bernasconi et al.[2] (Electronic Supplement) were used. They comprised a depth of 5-10 cm and did not include the youngest soil (0 yrs). The samples used in the present study were taken in direct proximity to the sites probed by Bernasconi et al.[2], comprising a slightly greater depth of 5- 20 cm.

	Soil age (yrs)						
	7	15	65	70	80	110	120
				wt.-%			
Na	3.0	1.9	2.8	2.7	2.8	2.2	2.8
Fe	1.4	1.3	1.2	1.9	1.8	1.5	0.9
O	47.7	50.4	48.4	48.7	50.5	50.6	49.0
K	3.6	3.4	2.8	3.2	3.4	2.9	3.1
Ca	0.7	0.4	0.7	1.0	0.6	0.6	0.7
Si	35.1	34.1	35.1	33.7	31.8	33.7	35.1
Mg	0.4	0.3	0.5	0.7	0.6	0.4	0.2
Al	8.2	8.2	8.5	8.3	8.5	8.3	8.2
			(-)				
CIA	0.52	0.58	0.55	0.53	0.53	0.58	0.55

Table S1: Bulk element contents as function of soil age (except 0 yrs) for a depth of 5-10 cm, adopted from the study of Bernasconi et al.[2] (Electronic Supplement). Additionally given is the chemical index of alteration (CIA) as a measure for intensity of weathering[2].

Determination of layer thickness *t*

Inelastic scattering of photoelectrons (PE) causes kinetic energy (E_K) shifts and limits the emission depth of counted PE. The mean free path λ, (nm) gives the distance a PE can travel through solid matter without losing its element-specific BE. Seah & Dench[3] compiled a database to calculate λ for different materials. The element content in combination with λ thus is used to determine the thickness of surface layers [4,5]. The approach of Pantano & Wittberg[5] originally was developed for glass fibers coated by organosilanes and has the advantage of an easy-to-use formula. The layer thickness t (nm) here is determined from the content of an element X occurring only in the underlying material but still is detected in the spectra of the coated surface and λ of the PE used for calculation. The content of X of the non-coated surface is derived from regression analysis with a PE with similar λ of an element Y occurring only in the coating.

The mean free path is determined from E_k as[4]

$$\lambda = 0.087 E_K^{\,0.5} \qquad (1)$$

E_k = kinetic energy (eV)

The layer thickness t then is[4]

$$t = -\lambda_X \ln\left(\frac{I_X}{I_{X^*}}\right)\frac{2}{\pi} \quad . \qquad (2)$$

I_X = content of element X determined for the coated surface (at.-%)
I_{X*} = content of element X of the non-coated surface as determined from regression analysis with element Y (at.-%)

The term $2/\pi$ was added by Pantano & Wittberg[5] to correct for the curved surface of the analyzed glass fibers and was used here as well to consider the roundish surface of soil particles. The regression analyses, i.e., Si *vs*. C and Al *vs*. C (Fig. S3), resulted for 0 yrs in greater calculated Si and Al contents than measured (18.4 at- % vs. 14.5 at.-% and 7.6 at.-% *vs*. 5.9 at.- %, respectively; Table 1, main text). As the regression assumes bare mineral surfaces for the initial stage of soil formation (i.e., 0 yrs) this result fits with the actually measured C content (Table 1, main text) that indicates C components already present at this stage (see main text).

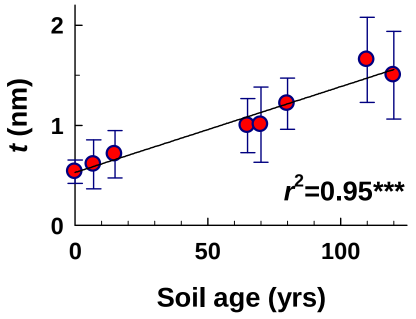

Figure S4. Mean layer thickness t (determined from t_{Si} and t_{Al}; n = 6) as function of soil age. The line represents a linear regression fit. Significance level: *** P < 0.001.

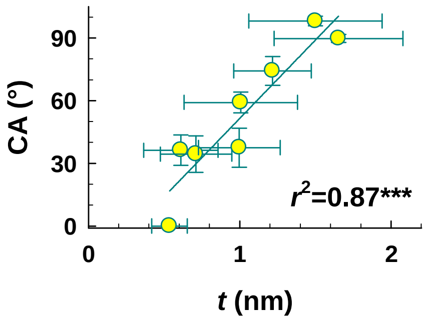

Figure S5. The contact angle (CA) as function of the mean layer thickness t (n = 6). The line represents a linear regression fit. Significance level: *** P < 0.001.

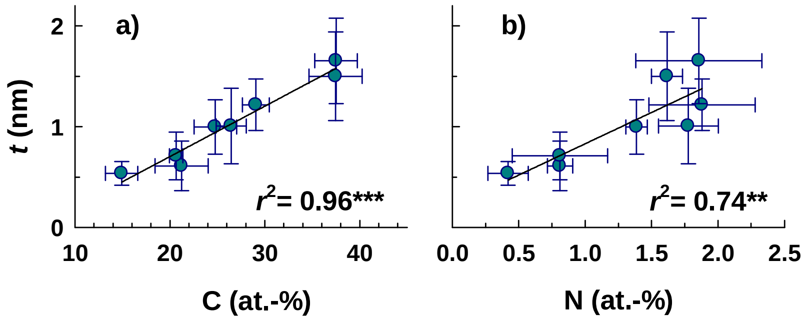

Figure S6. Mean layer thickness t (n = 6) as function of surface C (a) and N (b) content. The lines represent linear regression fits. Significance levels: *** P < 0.001; ** P < 0.01.

Differentiation between O bound to C (O_C) and O bound to mineral-derived cations (O_{cation})

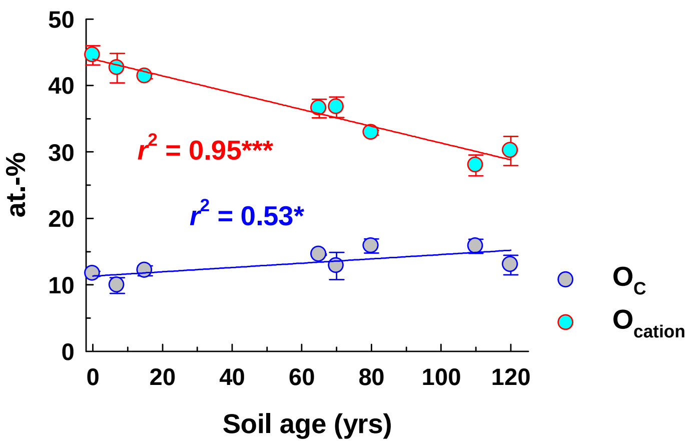

Figure S7. Content of O bound to C (O_C) and O bound to mineral-derived cations (O_{cation}) as function of soil age. The lines represent linear regression fits. Significance levels: * $P < 0.05$, *** $P < 0.001$.

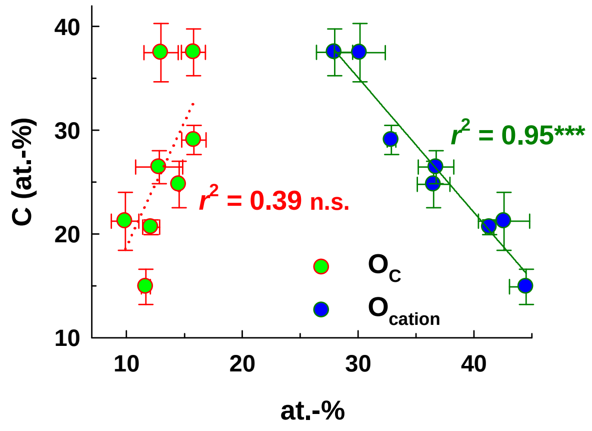

Figure S8. Relationship between C and O bound to C (O_C) and C and O bound to mineral-derived cations (O_{cation}). The line represents a linear regression fit. Significance level: *** $P < 0.001$. The dotted line added for O_c (no significant correlation with C, $P > 0.05$) indicates the general trend.

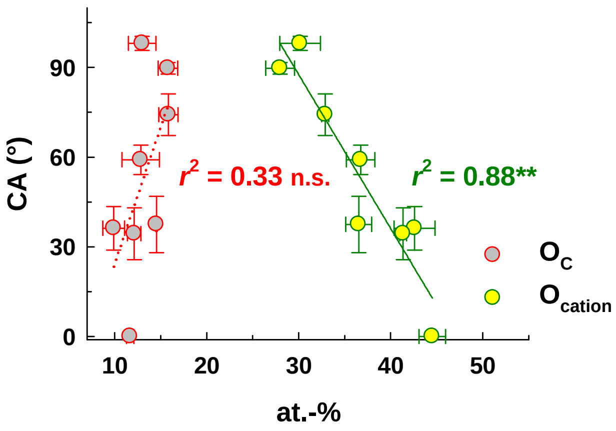

Figure S9. The contact angle (CA) as function of the amount of O bound to C (O_C) and O bound to mineral-derived cations (O_{cation}). The solid line represents a linear regression fit. Significance level: ** $P < 0.01$. The dotted line added for O_C (no significant correlation with CA, $P > 0.05$) indicates the general trend.

Characterization of the C species present by a general differentiation of total C in polar (C_p) and non-polar (C_{np}) C species

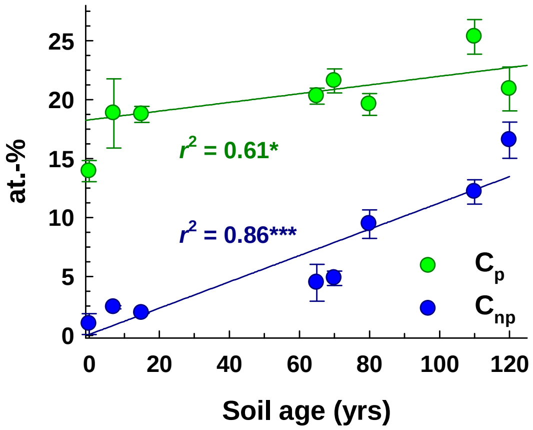

Figure S10. The amounts of polar (C_p) and non-polar (C_{np}) C species as function of soil age. The lines represent linear regression fits. Significance levels: * $P < 0.05$; *** $P < 0.001$. The amounts of C_p and C_{np} were determined by least squares fitting of the C 1s peak[6,7].

Remark: The amount of C_p basically only increased between 0 and 7 yrs, along with a distinct increase of total C content and the transition of the wetting properties from hydrophilic to subcritically water repellent (s. Table 1, main text). Considering only the interval between 7 and 120 yrs, C_p content did not show a significant correlation with soil age (r^2 = 0.49, P> 0.05).

Full width at half maximum (FWHM): comparison between ideal (smooth) and non-ideal (rough) surfaces

XPS analysis sensu stricto (like contact angle analysis) only is applicable to smooth (“ideal”) surfaces as surface roughness can affect the intensity of the XPS signal[8,9]. As an estimate for a possible influence of roughness, the Si 2p photoelectron´s full width at half maximum (FWHM) from smooth glass slides was used as a reference and compared with the respective Si 2p FWHM of rough surfaces, comprising the samples of this study and further soil and mineral samples ranging in texture from clay to coarse sand. The mean Si 2p FWHM of the glass surfaces was 3.05 eV ±0.13 (n = 6), the mean Si 2p FWHM of all other surfaces tested was 2.95 eV ±0.38 (n = 183). The absolute values thus were very similar, although the Mann-Whitney rank sum test indicated a statistically significant difference between both groups. However, the meaning of this result probably is limited given the considerable dissimilarity in the number of data points (6 *vs*. 183, derived from two and 61 samples, respectively) and thus should not challenge the use of XPS on rough surfaces, especially when referring to the main elements. However, roughness may be of influence for the detection and quantification of elements that are not evenly distributed and are present in only low amounts as must be assumed for P in case of the chronosequence samples. The increase in phospholipid fatty acids (PFFA) with soil age[10] thus could not be related to P content.

References

(1) Okada, K., Kameshima, Y. & Yasumori, A. Chemical shifts of silicon X-ray photoelectron spectra by polymerization structures of silicates. *J. Am. Ceram. Soc*. **81**, 1970–1972 (1998).

(2) Bernasconi, S. M. et al. Chemical and biological gradients along the Damma glacier soil chronosequence, Switzerland. *Vadose Zone J.* **10**, 867–883 (2011).

(3) Seah, M P. & Dench, W. A. Quantitative electron spectroscopy of surfaces: A standard data base for electron inelastic mean free paths in solids. *Surf. Interface Anal*. **1**, 2–11 (1979).

(4) Cole, D. A. et al. SiO_2 thickness determination by X-ray photoelectron spectroscopy, Auger electron spectroscopy, secondary ion mass spectrometry, Rutherford backscattering, transmission electron microscopy, and ellipsometry. *J. Vac. Sci. Technol*. **18**, 440–444 (2000).

(5) Pantano, C. G. & Wittberg, T. N. XPS analysis of silane coupling agents and silane-treated E-glass fibers. *Surf. Interface Anal*. **15**, 498–501 (1990).

(6) Krueger, J., Böttcher, J., Schmunk, C. & Bachmann, J. Water repellency and chemical soil properties in a beech forest subsoil – Spatial variability and interrelations. *Geoderma* **271**, 50–62 (2016).

(7) Pronk, G. J. et al. Interaction of minerals, organic matter, and microorganisms during biogeochemical interface formation as shown by a series of artificial soil experiments. *Biol. Fertil. Soils* **53**, 9–22 (2017).

(8) De Bernardez, L. S., Perrón, J., Goldberg, E. C., Buitrago, R.H. The effect of surface roughness on XPS and AES. *Surf. Sci*. **139**, 541–548 (1984).

(9) Gunter, P. L. J., Gijzeman, O. I. J, Niemantsverdriet, J. W. Surface roughness effects in quantitative XPS: magic angle for determining overlayer thickness. *Appl. Surf. Sci.* **115**, 342–346 (1997).

(10) Schurig, C. et al. Microbial cell-envelope fragments and the formation of soil organic matter: a case study from a glacier forefield. *Biogeochemistry* **113**, 595–612 (2013).
